# 2,4-Dichloro-6-nitro­benzoic acid

**DOI:** 10.1107/S1600536808002560

**Published:** 2008-01-30

**Authors:** Hai-Lian Liu, Zhi-Qiang Du

**Affiliations:** a Department of Chemistry, Zhejiang University, Hangzhou 310027, People’s Republic of China

## Abstract

The title compound, C_7_H_3_Cl_2_NO_4_, was prepared by the reaction of 2,4-dichloro-6-nitro­toluene with 20% HNO_3_ solution at 430 K. The carboxyl and nitro groups are twisted by 82.82 (12) and 11.9 (2)°, respectively, with respect to the benzene ring. The crystal structure is stabilized by O—H⋯O hydrogen bonding between carboxyl groups and weak C—H⋯O hydrogen bonding between the nitro group and the benzene ring of an adjacent mol­ecule.

## Related literature

For general background, see: Jacobson (1997 ▶); Langer *et al.* (2006 ▶); Li & Zhu (2007 ▶).
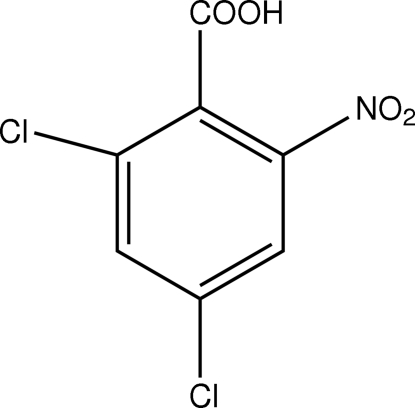


## Experimental

### 

#### Crystal data

C_7_H_3_Cl_2_NO_4_
*M* *_r_* = 236.00
Triclinic, 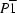

*a* = 4.6930 (7) Å
*b* = 7.5590 (11) Å
*c* = 13.0721 (19) Å
α = 97.120 (2)°
β = 95.267 (2)°
γ = 100.631 (2)°
*V* = 449.11 (11) Å^3^
*Z* = 2
Mo *K*α radiation
μ = 0.71 mm^−1^
*T* = 295 (2) K
0.40 × 0.30 × 0.20 mm

#### Data collection

Bruker SMART CCD area-detector diffractometer
Absorption correction: multi-scan (*SADABS*; Bruker, 2002 ▶) *T*_min_ = 0.765, *T*_max_ = 0.872
2415 measured reflections
1641 independent reflections
1457 reflections with *I* > 2σ(*I*)
*R*_int_ = 0.011

#### Refinement

*R*[*F* ^2^ > 2σ(*F* ^2^)] = 0.032
*wR*(*F* ^2^) = 0.086
*S* = 1.06
1641 reflections
127 parameters
H-atom parameters constrained
Δρ_max_ = 0.22 e Å^−3^
Δρ_min_ = −0.34 e Å^−3^

### 

Data collection: *SMART* (Bruker, 2002 ▶); cell refinement: *SAINT* (Bruker, 2002 ▶); data reduction: *SAINT*; program(s) used to solve structure: *SHELXS97* (Sheldrick, 2008 ▶); program(s) used to refine structure: *SHELXL97* (Sheldrick, 2008 ▶); molecular graphics: *ORTEP-3 for Windows* (Farrugia, 1997 ▶); software used to prepare material for publication: *WinGX* (Farrugia, 1999 ▶).

## Supplementary Material

Crystal structure: contains datablocks I, global. DOI: 10.1107/S1600536808002560/xu2399sup1.cif

Structure factors: contains datablocks I. DOI: 10.1107/S1600536808002560/xu2399Isup2.hkl

Additional supplementary materials:  crystallographic information; 3D view; checkCIF report

## Figures and Tables

**Table 1:** Hydrogen-bond geometry (Å, °)

*D*—H⋯*A*	*D*—H	H⋯*A*	*D*⋯*A*	*D*—H⋯*A*
O4—H4*A*⋯O3^i^	0.90	1.77	2.664 (2)	173
C3—H5⋯O2^ii^	0.93	2.56	3.453 (2)	160

Symmetry codes: (i) 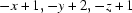; (ii) 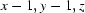.

**Fig. 1.**
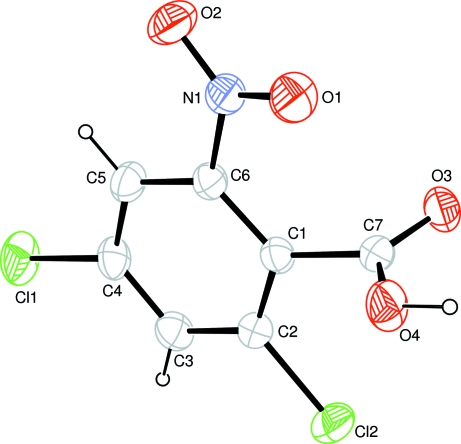
The molecular structure of the title compound with 40% probability displacement ellipsoids.

## supplementary crystallographic information

### Comment

Ortho-nitro aromatic acids have been used as intermediates of dyes, pharmaceuticals and agrochemicals (Jacobson, 1997; Langer *et al.*, 2006). The title compound is an important chemical intermediates of a kind of synthetic dyes, pharmaceuticals (Li & Zhu, 2007). Its crystal structure is reported here.

The molecular structure of the title compound is shown in Fig. 1. The molecule displays a non-planar structure. The carboxyl and nitro groups are twisted with respect to the benzene ring by 82.82 (12) and 11.9 (2)°, respectively. Within the carboxyl group, the O3—C7 bond distance is appreciably shorter than the O4—C7 bond distance (Table 1). The crystal structure is stabilized by O—H···O hydrogen bonding between carboxyl groups and weak C—H···O hydrogen bonding between nitro group and benzene ring of adjacent molecules (Table 2).

### Experimental

The title compound was prepared by a reaction of 2-nitro-4,6-dichlorotoluene (1 mmol) with 20% HNO_3_ solution (15 ml) in an autoclave at 430 K for 20 h. Single crystals suitable for X-ray data collection were obtained by recrystallization from a methanol solution.

### Refinement

Carboxyl H atom was located in a difference Fourier map and refined as riding in as-found relative position with *U*_iso_(H) = 1.5*U*_eq_(O). Other H atoms were placed in calculated positions with C—H = 0.93 Å and refined in riding mode, *U*_iso_(H) = 1.2*U*_eq_(C).

### Crystal data

**Table:**

C_7_H_3_Cl_2_NO_4_	*Z* = 2
*M**_r_* = 236.00	*F*_000_ = 236
Triclinic, *P*1	*D*_x_ = 1.745 Mg m^−^^3^
Hall symbol: -P 1	Mo *K*α radiation λ = 0.71073 Å
*a* = 4.6930 (7) Å	Cell parameters from 2069 reflections
*b* = 7.5590 (11) Å	θ = 2.8–27.5º
*c* = 13.0721 (19) Å	µ = 0.71 mm^−^^1^
α = 97.120 (2)º	*T* = 295 (2) K
β = 95.267 (2)º	Prism, colorless
γ = 100.631 (2)º	0.40 × 0.30 × 0.20 mm
*V* = 449.11 (11) Å^3^	

### Data collection

**Table:**

Bruker SMART CCD area-detector diffractometer	1641 independent reflections
Radiation source: fine-focus sealed tube	1457 reflections with *I* > 2σ(*I*)
Monochromator: graphite	*R*_int_ = 0.011
*T* = 295(2) K	θ_max_ = 25.5º
φ and ω scans	θ_min_ = 2.8º
Absorption correction: multi-scan(SADABS; Bruker, 2002)	*h* = −5→5
*T*_min_ = 0.765, *T*_max_ = 0.872	*k* = −9→9
2415 measured reflections	*l* = −15→15

### Refinement

**Table:**

Refinement on *F*^2^	Secondary atom site location: difference Fourier map
Least-squares matrix: full	Hydrogen site location: inferred from neighbouring sites
*R*[*F*^2^ > 2σ(*F*^2^)] = 0.032	H-atom parameters constrained
*wR*(*F*^2^) = 0.087	*w* = 1/[σ^2^(*F*_o_^2^) + (0.0463*P*)^2^ + 0.1228*P*] where *P* = (*F*_o_^2^ + 2*F*_c_^2^)/3
*S* = 1.06	(Δ/σ)_max_ < 0.001
1641 reflections	Δρ_max_ = 0.22 e Å^−^^3^
127 parameters	Δρ_min_ = −0.34 e Å^−^^3^
Primary atom site location: structure-invariant direct methods	Extinction correction: none

### Special details

**Table:**

Geometry. All e.s.d.'s (except the e.s.d. in the dihedral angle between two l.s. planes) are estimated using the full covariance matrix. The cell e.s.d.'s are taken into account individually in the estimation of e.s.d.'s in distances, angles and torsion angles; correlations between e.s.d.'s in cell parameters are only used when they are defined by crystal symmetry. An approximate (isotropic) treatment of cell e.s.d.'s is used for estimating e.s.d.'s involving l.s. planes.
Refinement. Refinement of *F*^2^ against ALL reflections. The weighted *R*-factor *wR* and goodness of fit *S* are based on *F*^2^, conventional *R*-factors *R* are based on *F*, with *F* set to zero for negative *F*^2^. The threshold expression of *F*^2^ > σ(*F*^2^) is used only for calculating *R*-factors(gt) *etc*. and is not relevant to the choice of reflections for refinement. *R*-factors based on *F*^2^ are statistically about twice as large as those based on *F*, and *R*- factors based on ALL data will be even larger.

### Fractional atomic coordinates and isotropic or equivalent isotropic displacement parameters (Å^2^)

**Table:**

	*x*	*y*	*z*	*U*_iso_*/*U*_eq_	
Cl1	0.66419 (15)	0.23838 (7)	0.04930 (4)	0.0620 (2)	
Cl2	0.19170 (13)	0.45919 (8)	0.38873 (4)	0.0611 (2)	
N1	0.8796 (4)	0.9077 (2)	0.20929 (13)	0.0425 (4)	
O1	0.8119 (3)	1.03193 (18)	0.26405 (12)	0.0542 (4)	
O2	1.0647 (4)	0.9276 (2)	0.15015 (13)	0.0651 (5)	
O3	0.7264 (3)	0.86356 (19)	0.45830 (10)	0.0456 (3)	
O4	0.3076 (3)	0.9004 (2)	0.37628 (11)	0.0540 (4)	
H4A	0.2886	0.9850	0.4282	0.081*	
C1	0.5569 (4)	0.6891 (2)	0.29482 (13)	0.0335 (4)	
C2	0.4126 (4)	0.5107 (3)	0.29348 (14)	0.0388 (4)	
C3	0.4410 (4)	0.3708 (2)	0.21800 (15)	0.0428 (5)	
H5	0.3413	0.2525	0.2182	0.051*	
C4	0.6193 (4)	0.4107 (3)	0.14305 (15)	0.0413 (4)	
C5	0.7657 (4)	0.5858 (3)	0.14027 (15)	0.0410 (4)	
H3	0.8852	0.6117	0.0889	0.049*	
C6	0.7292 (4)	0.7212 (2)	0.21587 (14)	0.0346 (4)	
C7	0.5340 (4)	0.8318 (2)	0.38368 (14)	0.0352 (4)	

### Atomic displacement parameters (Å^2^)

**Table:**

	*U*^11^	*U*^22^	*U*^33^	*U*^12^	*U*^13^	*U*^23^
Cl1	0.0958 (5)	0.0399 (3)	0.0486 (3)	0.0164 (3)	0.0174 (3)	−0.0113 (2)
Cl2	0.0633 (4)	0.0609 (4)	0.0532 (3)	−0.0092 (3)	0.0264 (3)	0.0033 (3)
N1	0.0503 (9)	0.0346 (8)	0.0403 (9)	0.0024 (7)	0.0099 (7)	0.0021 (7)
O1	0.0708 (10)	0.0309 (7)	0.0605 (9)	0.0096 (6)	0.0189 (7)	−0.0021 (6)
O2	0.0816 (11)	0.0478 (9)	0.0627 (10)	−0.0061 (8)	0.0389 (9)	0.0017 (7)
O3	0.0431 (7)	0.0502 (8)	0.0388 (7)	0.0098 (6)	−0.0012 (6)	−0.0080 (6)
O4	0.0438 (8)	0.0655 (10)	0.0506 (8)	0.0230 (7)	0.0038 (6)	−0.0162 (7)
C1	0.0319 (9)	0.0341 (9)	0.0326 (9)	0.0062 (7)	0.0029 (7)	−0.0017 (7)
C2	0.0370 (10)	0.0418 (10)	0.0349 (9)	0.0026 (8)	0.0064 (8)	0.0013 (8)
C3	0.0490 (11)	0.0306 (9)	0.0442 (11)	0.0003 (8)	0.0042 (9)	0.0003 (8)
C4	0.0531 (11)	0.0346 (10)	0.0347 (10)	0.0106 (8)	0.0054 (8)	−0.0047 (7)
C5	0.0496 (11)	0.0385 (10)	0.0354 (10)	0.0088 (8)	0.0131 (8)	0.0008 (8)
C6	0.0388 (9)	0.0301 (9)	0.0330 (9)	0.0040 (7)	0.0056 (7)	0.0007 (7)
C7	0.0327 (9)	0.0372 (10)	0.0341 (9)	0.0048 (7)	0.0077 (7)	−0.0009 (7)

### Geometric parameters (Å, °)

**Table:**

Cl1—C4	1.7309 (18)	C1—C2	1.390 (3)
Cl2—C2	1.7277 (19)	C1—C7	1.510 (2)
N1—O2	1.216 (2)	C2—C3	1.388 (3)
N1—O1	1.217 (2)	C3—C4	1.372 (3)
N1—C6	1.472 (2)	C3—H5	0.9300
O3—C7	1.235 (2)	C4—C5	1.381 (3)
O4—C7	1.266 (2)	C5—C6	1.377 (2)
O4—H4A	0.8961	C5—H3	0.9300
C1—C6	1.386 (3)		
			
O2—N1—O1	124.25 (16)	C3—C4—C5	121.65 (17)
O2—N1—C6	117.96 (16)	C3—C4—Cl1	119.64 (15)
O1—N1—C6	117.79 (16)	C5—C4—Cl1	118.71 (15)
C7—O4—H4A	117.5	C6—C5—C4	117.98 (18)
C6—C1—C2	116.50 (16)	C6—C5—H3	121.0
C6—C1—C7	124.11 (16)	C4—C5—H3	121.0
C2—C1—C7	119.26 (16)	C5—C6—C1	123.15 (17)
C3—C2—C1	122.11 (17)	C5—C6—N1	117.00 (16)
C3—C2—Cl2	118.41 (15)	C1—C6—N1	119.84 (15)
C1—C2—Cl2	119.48 (14)	O3—C7—O4	126.27 (17)
C4—C3—C2	118.60 (17)	O3—C7—C1	117.77 (15)
C4—C3—H5	120.7	O4—C7—C1	115.83 (15)
C2—C3—H5	120.7		

### Hydrogen-bond geometry (Å, °)

**Table:**

*D*—H···*A*	*D*—H	H···*A*	*D*···*A*	*D*—H···*A*
O4—H4A···O3^i^	0.90	1.77	2.664 (2)	173
C3—H5···O2^ii^	0.93	2.56	3.453 (2)	160

Symmetry codes: (i) −*x*+1, −*y*+2, −*z*+1; (ii) *x*−1, *y*−1, *z*.
